# Supplementary material for: ADAM17 Inhibition Increases the Impact of Cisplatin Treatment in Ovarian Cancer Spheroids
Source: Cancers (Basel). 2021 Apr 23;13(9):2039. doi: 10.3390/cancers13092039 (PMC8122950; doi:10.3390/cancers13092039)
Supplement: Supplementary file 1 [file cancers-13-02039-s001.zip › cancers-1156043-supplementary-for pub.pdf]

cancers

ADAM17 Inhibition Increases the Impact of Cisplatin Treatment in Ovarian Cancer Spheroids

Nina Hedemann, Andreas Herz, Jan Hendrik Schiepanski, Jan Dittrich, Susanne Sebens, Astrid Dempfle, Julia Feuerborn, Christoph Rogmans, Nils Tribian, Inken Flörkemeier, Jörg Weimer, Sandra Krüger, Nicolai Maass and Dirk O. Bauerschlag

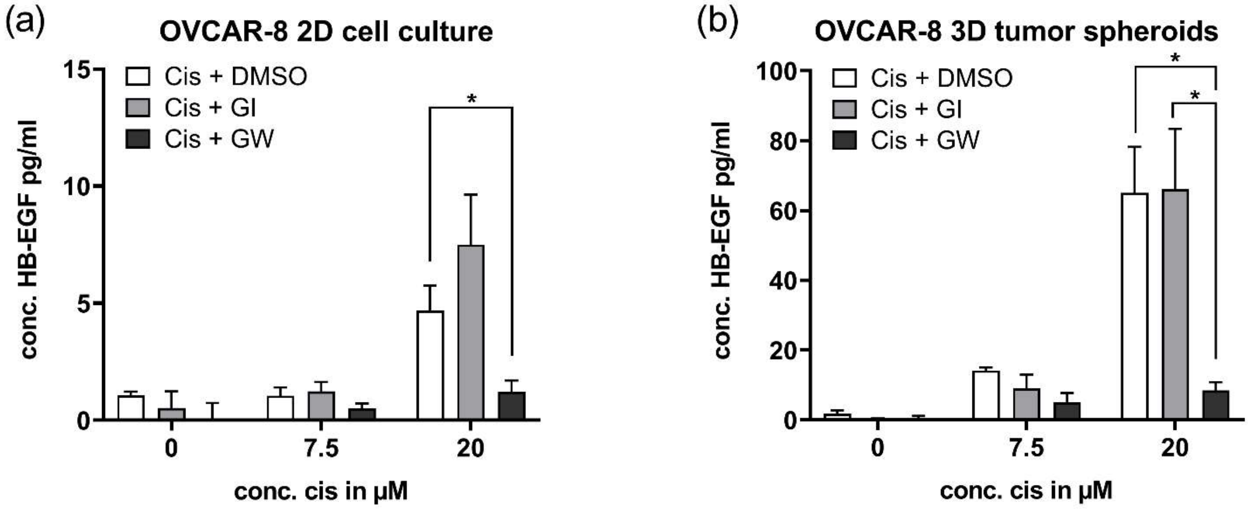

Figure S1: ADAM17 substrate release is enhanced by cisplatin treatment and inhibited by GW280264X in 2D cell cultures and 3D tumor spheroids.

OVCAR-8 cells were treated for 48 h with the indicated concentrations of cisplatin (cis) and the following inhibitors: ADAM17 activity was inhibited using 3 µM GW280264X (GW) and ADAM10 using 3 µM GI254023X (GI). DMSO was used as a solvent control. Heparin-binding EGF (HB-EGF) in supernatants was measured using ELISA, as a surrogate for ADAM17 activity. Induced HB-EGF release is abrogated by GW but not by GI. This effect is prominent in two dimensional (2D) monolayer cultures (a) and three dimensional (3D) spheroids (b). Displayed is the mean (+ SEM) of three biological replicates. Based on Shapiro-Wilk normality test, values of three biological replicates were analyzed by ANOVA following Tukey´s multiple comparison test. Comparison between HB-EGF levels induced by DMSO or GI + cisplatin vs. GW + cisplatin: p = significance, *($p<0.05$).
